# Interleukin-6 Mediates Angiotensinogen Gene Expression during Liver Regeneration

**DOI:** 10.1371/journal.pone.0067868

**Published:** 2013-07-03

**Authors:** Hong-Shiee Lai, Wen-Hsi Lin, Shuo-Lun Lai, Hao-Yu Lin, Wen-Ming Hsu, Chia-Hung Chou, Po-Huang Lee

**Affiliations:** Department of Surgery, National Taiwan University Hospital and National Taiwan University College of Medicine, Taipei, Taiwan; Wayne State University, United States of America

## Abstract

**Background:**

Angiotensinogen is the precursor of angiotensin II, which is associated with ischemia-reperfusion injury. Angiotensin II reduces liver regeneration after hepatectomy and causes dysfunction and failure of reduced-size liver transplants. However, the regulation of angiotensinogen during liver regeneration is still unclear.

**Aims:**

To investigate the regulation of angiotensinogen during liver regeneration for preventing angiotensin II-related ischemia-reperfusion injury during liver regeneration.

**Methods:**

A mouse *in vitro* partial hepatectomy animal model was used to evaluate the expression of interleukin-6 (IL-6) and angiotensinogen during liver regeneration. Serum IL-6 and angiotensinogen were detected by enzyme immunoassay (EIA). Angiotensinogen mRNA was detected by RT-PCR. Tissue levels of angiotensinogen protein were detected by Western blot analysis. Primary cultures of mouse hepatocytes were used to investigate IL-6-induced angiotensinogen. Chemical inhibitors were used to perturb signal transduction pathways. Synthetic double-stranded oligodeoxynucleotides (ODNs) were used as ‘decoy’ cis-elements to investigate transcription. Ki 67 staining and quantification were used to verify liver regeneration.

**Results:**

In the *in vivo* model, the levels of serum IL-6 and angiotensinogen correlated. In the *in vitro* model, IL-6 transcriptionally regulated angiotensinogen expression. Additionally, IL-6 mediated angiotensinogen expression through the Janus kinase (JAK)/signal transducer and activator of transcription 3 (STAT3) and JAK/p38 signaling. Decoy ODN analyses revealed that STAT3 and nuclear factor-kB (NF-kB) also played critical roles in the transcriptional regulation of angiotensinogen by IL-6. IL-6-mediated signaling, JAK2, STAT3 and p38 inhibitors reduced angiotensinogen expression in the partially hepatectomized mice.

**Conclusion:**

During liver regeneration, IL-6-enhanced angiotensinogen expression is dependent on the JAK/STAT3 and JAK/p38/NF-kB signaling pathways. Interruption of the molecular mechanisms of angiotensinogen regulation may be applied as the basis of therapeutic strategies for preventing angiotensin II-related ischemia-reperfusion injury during liver regeneration.

## Introduction

Liver regeneration occurs after a loss of liver mass or liver injury, such as that resulting from the resection of liver tumors or trauma repair [1], [2]. Liver regeneration is widely studied by mimicking such clinical conditions via partial hepatectomy in rodents. The reproducibility of partial hepatectomy has made it the preferred approach for studies of liver regeneration. In rats and mice, partial hepatectomy triggers a sequence of regeneration events from the first 30 minutes to 7 days after the procedure. A large number of genes with either new or increased expression in the regenerating liver have been identified and categorized into several phases of activity, including immediate-early, delayed, cell cycle, and DNA replication and mitosis genes [3], [4].

Angiogenesis, an important process for tissue growth, is also essential for liver regeneration [5]. During liver regeneration, several factors are involved in angiogenesis including plasminogen, vascular endothelial growth factor, and vascular endothelial cells [6]–[8]. On the other hand, apoptosis, a common form of cell death, occurs under both physiological and pathological conditions. Apoptosis and cell proliferation are complementary and account for the maintenance, growth, or degradation of a tissue. The regulation of apoptosis is important during liver regeneration [9], [10], and there is a fine balance between cell proliferation and apoptosis. Interleukin-6 (IL-6) is an important cytokine involved in liver regeneration. Hepatocyte DNA synthesis during liver regeneration is suppressed in mice carrying a homozygous deletion of the *IL-6* gene [11]. Moreover, IL-6 plays a crucial role in regulating the regeneration of hepatocytes after hepatitis or partial hepatectomy [12].

Angiotensinogen, an essential member in the rennin-angiotensin system, is responsible for hypertension [13], [14], and angiotensinogen is also associated with liver cirrhosis, portal hypertension and hepatic ischemia/reperfusion injury [15], [16]. Interestingly, angiotensinogen is related to both angiogenesis and apoptosis. Angiotensinogen inhibits angiogenesis by inducing apoptosis of endothelial cells [17], and it also enhances apoptosis of other cell types, including alveolar cells, cardiomyocytes and renal tubular cells [18]–[20]. This evidence suggests that angiotensinogen plays an essential role in the process of liver regeneration. Angiotensinogen serves as a reservoir for angiotensin I, which is cleaved from the N-terminal by the enzyme renin and is then converted into angiotensin II. Angiotensin II-related ischemia-reperfusion reduces liver regeneration after hepatectomy and is also a cause of dysfunction and failure of reduced-size liver transplants [21]. In this study, we defined the molecular regulatory effects of IL-6 on angiotensinogen during liver regeneration. IL-6 mediated angiotensinogen gene expression during liver regeneration after partial hepatectomy through the Janus kinase (JAK)/signal transducer and activator of transcription 3 (STAT3) or JAK/p38/NF-kB signaling pathway.

## Materials and Methods

### Animals

Male imprinting control region (ICR) mice (purchased from Charles River, Osaka, Japan) weighing approximately 30 g each were used in the experiments. All mice were randomly assigned to two groups that were subjected to either 70% partial hepatectomy or a sham operation. Mice were further divided into eight subgroups that were killed either preoperatively (0 h) or 2, 4, 6, 12, 24 hours, 3 and 7 days postoperatively. All the animal care and handling procedures and experimental protocols were approved by the Committee of Experimental Animal Management at College of Medicine, National Taiwan University.

### Surgical Procedures

All mice were anesthetized by inhalation of isoflurane [2-chloro-2- (difluoromethoxy)-1,1,1-trifluoro-ethane]. Partial hepatectomy was then performed through a midline laparotomy by aseptically extirpating the median and left lateral lobes, accounting for approximately 70% of the original liver, according to the procedure of Higgins and Anderson [22]. The removed liver sample was immediately weighed and prepared for Western blot analysis. Laparotomies with manipulation of the liver were performed as the sham operation. At sacrifice, the remnant liver was excised and weighed. The original liver weight was estimated retrospectively based on the excised liver weight after 70% PH. For each time point, the ratio of remnant liver weight to the estimated original liver weight was calculated as a percentage value.

### Measurement of IL-6 and Angiotensinogen Serum Levels

The serum levels of IL-6 and angiotensinogen were determined by enzyme immunoassay (R&D Systems, Minneapolis, MN, USA for IL-6 and Immuno-Biological Laboratories Co., Ltd., Aramachi, JAPAN for angiotensinogen) at different time points after partial hepatectomy or the sham operation.

### Angiotensinogen mRNA Expression Determination

Total RNA was isolated from remnant liver tissue using RNAzol B reagent (Invitrogen, San Diego, CA) according to the manufacturer’s instructions. cDNA was prepared from 2 µg of total RNA with random hexamer primers according to the cDNA synthesis ImProm-II protocol (Promega, Madison, USA). Specific oligonucleotide primer pairs for angiotensinogen were used in the reverse transcription-polymerase chain reaction.

### Western Blotting

The tissue lysates were centrifuged at 1,000×g for 10 minutes at 4°C. Protein concentrations were measured using the Bio-Rad protein assay (Hercules, CA). Then, 50-µg protein samples were separated using SDS-PAGE, transferred onto polyvinylidene difluoride membranes and immuno-blotted with various antibodies. Results were quantified by densitometry (Bio-Rad GS-800 densitometer).

### Antibodies and Chemical Inhibitors

Akt (protein kinase B) antibody, Akt phosphorylations, ERK (extracellular signal-regulated kinase), ERK phosphorylations, p38, p38 phosphorylations, c-Jun N-terminal kinase (JNK), JNK phosphorylations, epidermal growth factor receptor (EGFR), EGFR phosphorylations, STAT3, STAT3 phosphorylations, and NF-kB p65 were purchased from Santa Cruz Biotechnology (Santa Cruz, CA). Anti-β-actin antibody was purchased from Abcam (Cambridge, MA). Chemical inhibitors of LY294002, PD98059, SB203580, AG1478, AG490, and SP600125 were purchased from Sigma Chemical Company (St. Louis, MO). STAT3 inhibitor, S31–201, was purchased from Calbiochem (San Diego, CA).

### Culture of Mouse Hepatocytes

The mouse hepatocytes were collected from regenerated mouse livers. The cell suspensions were layered onto 10 ml of Ficoll-Paque PLUS (GE Healthcare Lifesciences). After centrifugation at 450 × g for 15 minutes, the cells in interphase were collected. The cells were treated with 80 IU/ml hyaluronidase in 1 ml of RPMI-1640 medium (Gibco®) for 10 minutes. The cells were washed and suspended in RPMI-1640 medium containing 10% fetal bovine serum, 100 U/ml penicillin, 0.1 mg/ml streptomycin, and 0.25 mg/ml amphotericin B. The cells were then incubated at 37°C in a humidified atmosphere with 5% CO_2_.

### Double-stranded Oligodeoxynucleotide Decoys

This study used synthetic, double-stranded oligodeoxynucleotides (ODNs) as ‘decoy’ cis-elements to block the binding of nuclear factors to the promoter regions of targeted genes, thus inhibiting gene transactivation. The following sequences of the phosphorothioate ODN were utilized: Scramble decoy ODN: 5′-TTGCCGTACCTGACTTAGCC-3′; C/EBP decoy ODN: 5′-TGCAGATTGCGCAATCTGCA-3′; AP-1 decoy ODN: 5′-CGCTTGATGACTCA GCCGGAA-3′; NF-kB decoy ODN: 5′-CCTTGAAGGGATTTCCCTCC-3′; CRE decoy ODN: 5′-TGACGTCATGACGTCATGACGTCA-3′; Sp-1 decoy ODN: 5′-GTGGGTGGGGCTGGAACAT-3′; and STAT3 decoy ODN: 5′-CATTTCCCGTAA ATC-3′. For transfection of hepatocytes, the decoy or scrambled decoy was mixed with the Transfast transfection reagent (Promega) for 15 minutes and then incubated with the cells in a serum-free medium.

### Immunohistochemical Assay

The slides were rehydrated in PBS for 15 min, and endogenous peroxidase was inhibited by exposure (10 min at room temperature) to a solution of 3% H_2_O_2_ in methanol. Samples were blocked (30 min at room temperature) using 5% nonfat milk in PBS. Slides then were incubated with Ki-67 (sc-7846, Santa Cruz Biotechnology, Inc.)http://www.scbt.com/login.html for 16 hours at 4°C. The peroxidase-conjugated secondary antibody was incubated for 1 hour at room temperature, and slides were developed by immersing them in 0.06% 3,3′-diaminobenzidine tetrahydrochloride (DAKO), followed by counterstaining with Gill's Hematoxylin V. Proliferative activity was defined by the Ki-67 staining index (SI). Nuclear staining of Ki-67 was considered positive. The Ki-67 SI was defined as the average percentage of positive nuclei from 10 high-power fields (400x ) counted.

### Statistical Analysis

In this study, each experiment was performed in triplicate, and all experiments were repeated at least three times on different occasions. Data were expressed as means ± standard deviations. Paired Student’s *t*-tests were used to evaluate statistically significant differences between the experimental groups and the control group in specified tests. *P*<0.05 was considered as the statistically significant cut-off value.

## Results

### Expression of Serum IL-6 and Angiotensinogen Levels had Similar Temporal Patterns during Liver Regeneration

**Figure 1:**
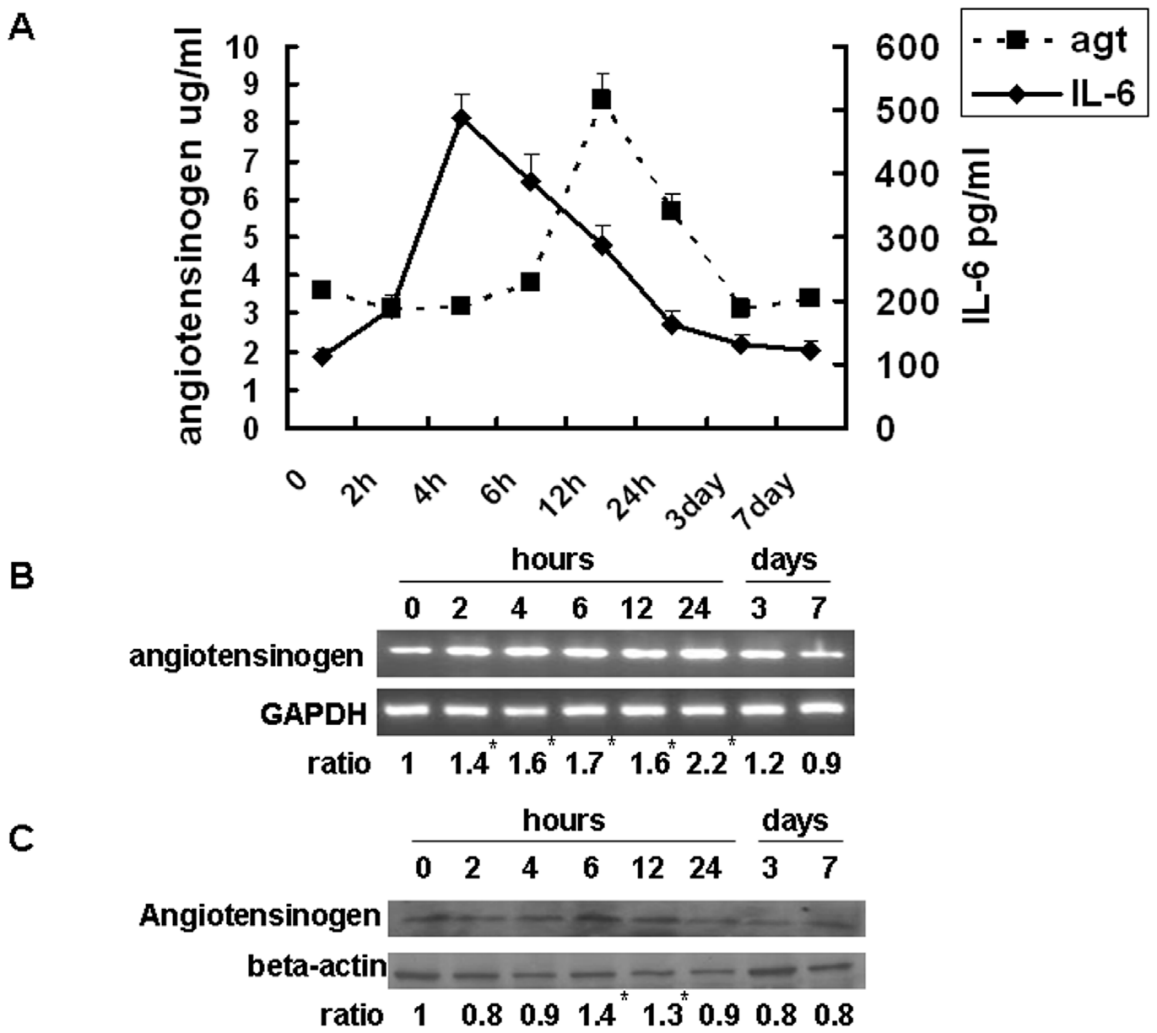
Serum IL-6 and angiotensinogen levels, angiotenisogen mRNA and protein expression in remnant livers after 70% partial hepatectomy in mice. (A) Serum angiotensinogen levels detected by enzyme immunoassay (n = 5/group). (B) Angiotensinogen mRNA detected by reverse transcription-polymerase chain reaction quantified by calculating the ratios of angiotensinogen/GAPDH. (C) Angiotensinogen protein expression detected by Western blot and quantified by calculating the ratios of angiotensinogen/ß-actin. The ratio in lane 1 is defined as 1. Comparison is between the time 0 group and specified time periods. *P<0.05, n = 5.

We evaluated the expression of IL-6 and angiotensinogen during liver regeneration after partial hepatectomy. Serum IL-6 levels peaked at 4 hours (486±38 pg/ml), while serum angiotensinogen levels peaked at 12 hours (8.6±0.67 µg/ml) and decreased at 24 hours (6.2±0.12 µg/ml) after partial hepatectomy (Fig. 1A). The *in vivo* partial hepatectomy-induced liver regeneration model showed that IL-6 expression began prior to that of angiotensinogen, but both had similar temporal patterns. Angiotensinogen mRNA in the remnant liver tissue was significantly upregulated from 2 to 24 hours after partial hepatectomy (Fig. 1B). Quantitative analysis of angiotensinogen protein levels in the remnant liver tissue revealed that angiotensinogen protein expression was significantly upregulated from 6 to 12 hours after partial hepatectomy (Fig. 1C).

### IL-6 induced Angiotensinogen Expression in Primary Cultures of Mouse Hepatocytes

**Figure 2:**
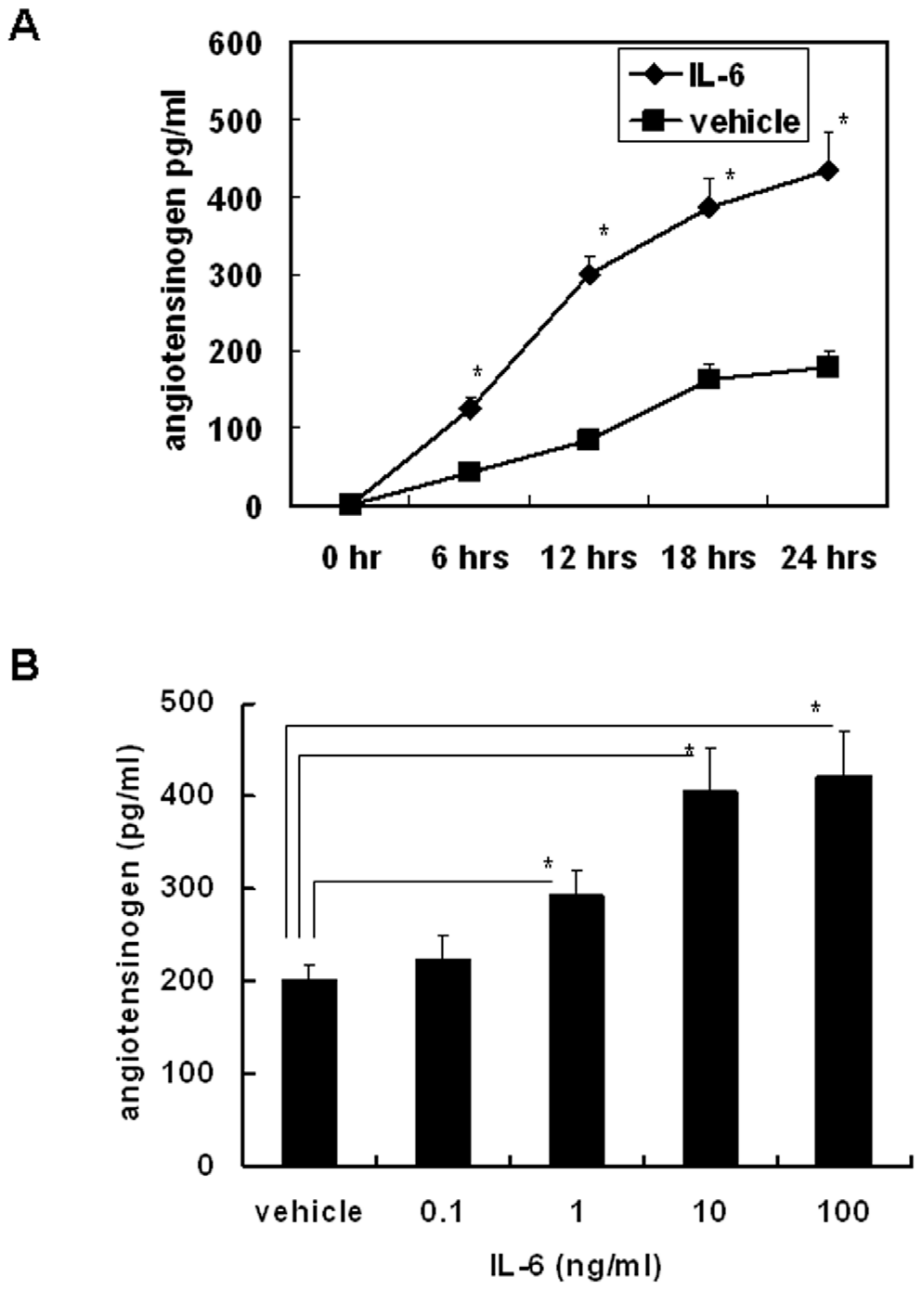
IL-6 induced angiotensinogen expression in primary cultures of mouse hepatocytes. (A) Time course of IL-6-induced (10 ng/ml) angiotensinogen protein expression as detected by enzyme immunoassay (n = 3/group). (B) Dose response of IL-6-induced angiotensinogen protein expression after 24 hours of culture, as detected by enzyme immunoassay. Comparisons are between the indicated groups. *P<0.05, n = 3.

To evaluate the induction effect of IL-6 on angiotensinogen expression, primary cultures of mouse hepatocytes were treated with 10 ng/ml of recombinant mouse IL-6. Angiotensinogen protein in the cell culture supernatant was measured at the specified times. Angiotensinogen levels were significantly higher in the IL-6-treated group than in the vehicle-treated group from 6 to 24 hours in the cell culture supernatant (Fig. 2A). To evaluate the dose response of IL-6 in inducing angiotensinogen protein expression, primary cultures of mouse hepatocytes were treated with different doses of recombinant mouse IL-6. After 24 hours of culture, angiotensinogen protein in the cell culture supernatants was measured. The data revealed that IL-6 significantly induced angiotensinogen protein expression at 1 to 100 ng/ml; the responses were also dose dependent in the range of 1 to 10 ng/ml (Fig. 2B).

### IL-6-mediated Angiotensinogen Expression is Dependent on the JAK/STAT3 and JAK/p38/NF-kB Signal Transduction Pathways

**Figure 3:**
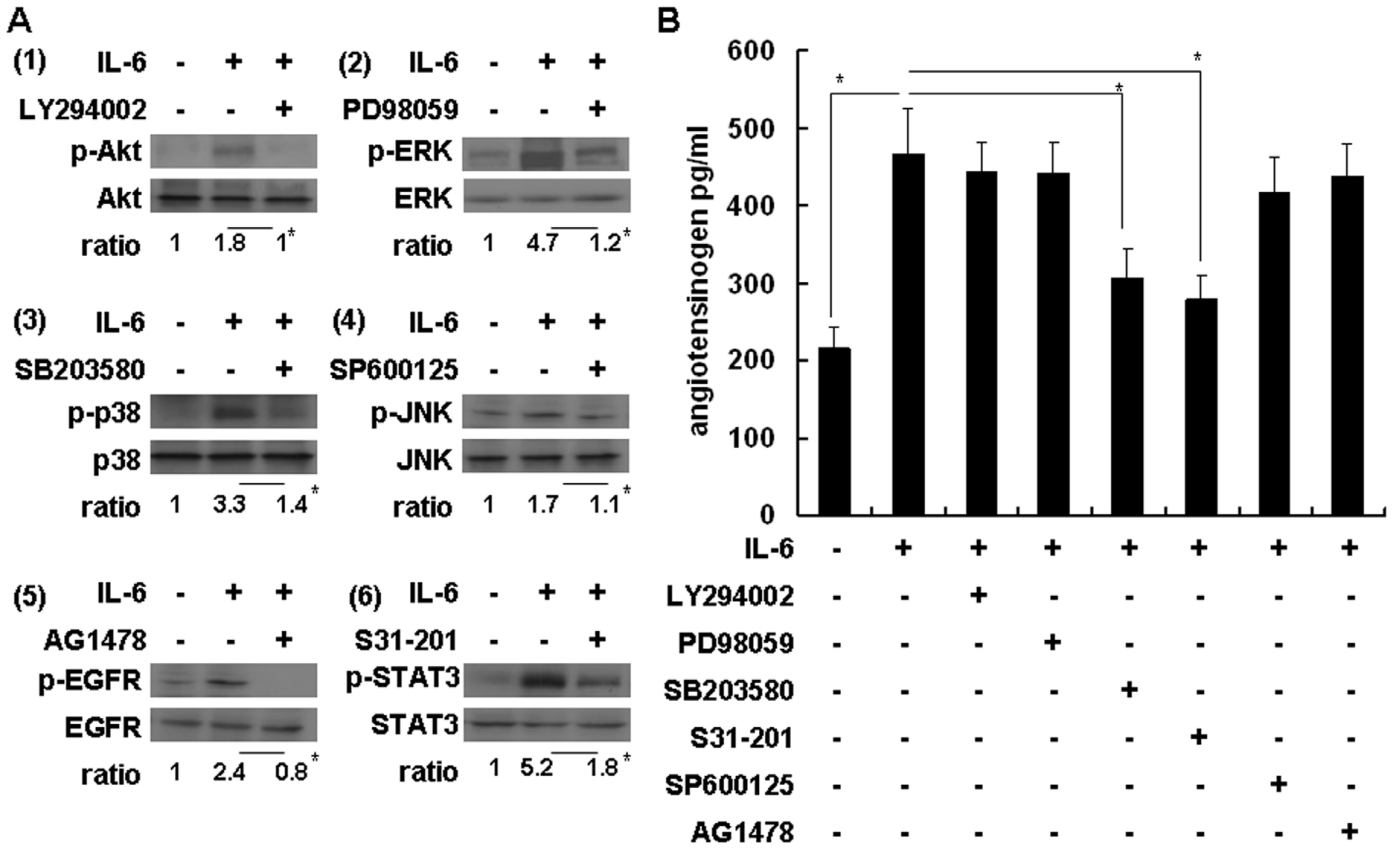
Signal transduction pathways involved in IL-6-induced angiotensinogen expression in primary cultures of mouse hepatocytes. (A) Inhibition effects of chemical inhibitors 1 to 6 on IL-6-activated signaling mediators detected by Western blotting and quantified by calculating the ratios of phosphorylated/non-phosphorylated protein forms. The ratio in lane 1 is defined as 1. Comparison is between lanes 2 and 3 in each group. *P<0.05, n = 3. (B) The effects of different chemical inhibitors on IL-6-induced angiotensinogen protein expression as detected by enzyme immunoassay. Comparison is between the indicated groups. *P<0.05, n = 3.

The molecular mechanisms of IL-6-induced angiotensinogen expression are not well defined. When IL-6 binds to IL-6Rα, the resultant complex associates with the signal-transducing membrane protein gp130, thereby, inducing its dimerization and the initiation of intracellular signaling via the JAK/STAT, MAPK family and PI3K pathways. We further investigated the signal transduction pathways involved in IL-6-induced angiotensinogen expression in primary cultures of mouse hepatocytes. Western blot analysis and the quantitative results revealed that the phosphatidylinositol 3-kinase inhibitor LY294002 (50 µM) significantly inhibited IL-6-induced Akt phosphorylation; the mitogen-activated protein kinase kinase inhibitor PD98059 (50 µg/ml) significantly inhibited IL-6-induced ERK phosphorylation; the mitogen-activated protein kinase p38 inhibitor SB203580 (5 µg/ml) significantly inhibited IL-6-induced p38 phosphorylation; the c-Jun N-terminal kinase (JNK) inhibitor SP600125 (20 µM) significantly inhibited IL-6-induced JNK phosphorylation; the signal transducer and activator of transcription 3 (STAT3) inhibitor S31–201 (10 µM) significantly inhibited IL-6-induced STAT3 phosphorylation; and the EGFR tyrosine kinase inhibitor AG1478 (20 µM) significantly inhibited IL-6-induced EGFR phosphorylation (Fig. 3A). We further investigated the inhibitory effects of different chemicals on IL-6-induced angiotensinogen protein expression. The results revealed that IL-6 significantly induced angiotensinogen protein expression (467±58 pg/ml), but pretreatment with SB203580 and S31–201 significantly inhibited IL-6-induced angiotensinogen protein expression (306±38 and 278±32 pg/ml, respectively) (Fig. 3B). Thus, the IL-6-mediated p38 and STAT3 signaling transduction pathways were critically involved in IL-6-induced angiotensinogen expression in primary cultures of mouse hepatocytes.

### STAT3 and NF-kB are Critical Transcription Factors Involved in IL-6-induced Angiotensinogen Expression in Mouse Hepatocytes

**Figure 4:**
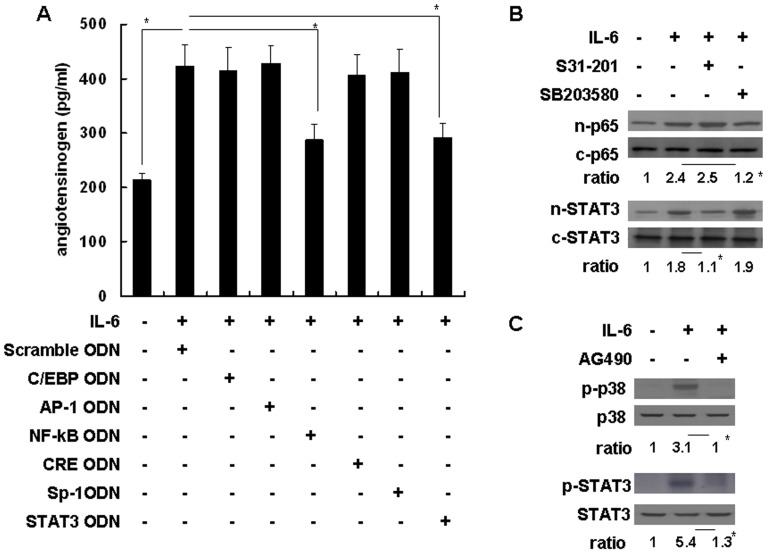
Transcription factors and their upstream signal mediators involved in IL-6-induced angiotensinogen expression in primary cultures of mouse hepatocytes. (A) Inhibition effects of different transcription factor oligodeoxynucleotide (ODN) decoys on IL-6-induced angiotensinogen protein expression as detected by enzyme immunoassay. Comparisons are between the indicated groups. *P<0.05, n = 3. (B) The effects of S31–201 and SB203580 on IL-6-induced STAT3 or NF-kB p65 subunit nuclear translocation were determined by Western blotting and quantified by calculating the ratio of n (nuclear)/c (cytosolic) proteins. The ratio in lane 1 is defined as 1. Comparisons are between lanes 2 and 3 or 4 in each group. *P<0.05, n = 3. (C) Effects of AG490 on IL-6-induced phospho-p38 (p-p38) and phospho-STAT3 (p-STAT3) were determined by Western blotting and quantified by calculating the ratio of phosphorylated/non-phosphorylated proteins. The ratio in lane 1 is defined as 1. Comparisons are between lanes 2 and 3 in each group. *P<0.05, n = 3.

To evaluate the transcription factors involved in the regulation of angiotensinogen by IL-6, synthetic double-stranded ODNs were used as ‘decoy’ cis-elements to block the binding of nuclear factors to the promoter regions of targeted genes, thus, inhibiting gene transactivation. In the promoter region of angiotensinogen, the oligonucleotides contained the consensus binding sequences for C/EBP, AP-1, NF-kB, CRE, Sp-1, and STAT3. Primary cultures of mouse hepatocytes were pretreated with different decoy ODNs (5 µM) for 24 hours prior to IL-6 (10 ng/ml) stimulation. IL-6 significantly induced angiotensinogen expression (423±38 pg/ml), while NF-kB and STAT3 decoy ODNs significantly inhibited IL-6-induced angiotensinogen expression (288±29 and 292±26 pg/ml, respectively) (Fig. 4A). We further clarified the effect of p38- and STAT3-mediated signaling on nuclear translocation of NF-kB and STAT3. Primary cultures of mouse hepatocytes were pretreated with the p38 inhibitor SB203580 or STAT3 inhibitor S31–201 for 1 hour prior to IL-6 stimulation; the nuclear and cytosolic proteins were collected after 1 hour. Quantitative analysis of the NF-kB p65 subunit and STAT3 revealed that p38 was significantly involved in IL-6-induced NF-kB nuclear translocation, and IL-6-induced STAT3 activation was significantly involved in STAT3 nuclear translocation. Furthermore, there was no crosstalk between IL-6-mediated p38 and STAT3 signaling (Fig. 4B). We also evaluated the role of Janus kinase (JAK) on IL-6-mediated STAT3 and p38 signaling using the JAK2-specific inhibitor AG490. Primary cultures of mouse hepatocytes were pretreated with AG490 for 1 hour prior to IL-6 stimulation. Western blot and quantitative analyses showed that JAK2 signaling was significantly involved in IL-6-activated p38 and STAT3 phosphorylation (Fig. 4C). Thus, IL-6-mediated JAK2/STAT3 and JAK2/p38/NF-kB signaling were required for proper IL-6-induced angiotensinogen expression in primary cultures of mouse hepatocytes.

### IL-6 Downstream Signaling JAK2, STAT3 and p38 Inhibitors Reduced Angiotensinogen Expression in the Partially Hepatectomized Mice

**Figure 5:**
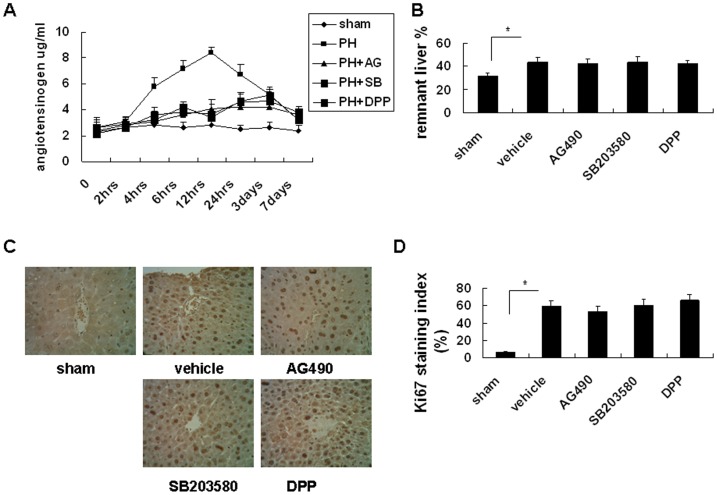
Effect of IL-6-related signaling inhibition on angiotensinogen protein expression and liver regeneration in remnant livers after 70% partial hepatectomy in mice. Mice were pretreated with chemical inhibitors of JAK2 (AG490, 10 mg/kg subcutaneously), p38 (intraperitoneal SB203580, 15 mg/kg), and STAT3 (intraperitoneal 5,15-DPP, 15 mg/kg) for 4 hours prior to partial hepatectomy. (A) Serum angiotensinogen levels of mice pretreated with different chemical inhibitors (AG490, SB203580, and 5,15-DPP) as detected by enzyme immunoassay. (B) Changes in the ratio of remnant to original liver weight after 70% partial hepatectomy. Remnant liver weight was estimated retrospectively from the excised liver weight after 70% PH. Data are presented as mean ± S.D., and comparisons were made between groups as indicated. *P<0.05. (C) Ki-67 staining of regenerated liver tissue sections of the indicated group. Magnification, 400x. (D) Quantification of Ki-67 staining. Data presented here are the quantification of Ki-67-positive nuclei per high-power field. Data are presented as mean percentage of positive nuclei ± S.D., and comparisons were made between groups as indicated. *P<0.05.

The plasma IL-6 concentration was reported to significantly and similarly associate with the systolic (SBP) and diastolic (DBP) blood pressure [23]. Furthermore, angiotensin II-associated hypertension was attenuated in interleukin-6 knockout mice [24]. However, the effects of IL-6 downstream signaling JAK2, STAT3 and p38 inhibitors on the *in vivo*, angiotensinogen expression and blood pressure in the partially hepatectomized mice are not clear. To confirm the effects of IL-6-mediated signaling on angiotensinogen expression *in vivo*, mice were pretreated with chemical inhibitors of JAK2 (AG490, 10 mg/kg subcutaneously), p38 (intraperitoneal SB203580, 15 mg/kg), and STAT3 (intraperitoneal 5,15-DPP, 15 mg/kg) for 4 hours prior to partial hepatectomy. The results revealed that all three chemical inhibitors significantly inhibited the expression of angiotensinogen from 12 to 24 hours after partial hepatectomy (Fig. 5A). To analyze other effects of these chemical inhibitors on liver regeneration, experimental data of the remnant liver revealed no significant inhibitory effect on liver regeneration (Fig. 5B) in 24 hours after partial hepatectomy; the effect was also confirmed by detecting the proliferation of hepatocytes by ki-67 staining (Fig. 5C). Furthermore, quantification of ki-67 staining (Fig. 5D) revealed that all three chemical inhibitors did not significantly inhibit liver regeneration after partial hepatectomy. These results demonstrate that IL-6-mediated signaling contributes to systemic angiotensinogen levels during liver regeneration after partial hepatectomy in the mouse model.

## Discussion

The phenomenon of liver regeneration after hepatic injury is unique [25]–[27]. Regulation of gene activation plays an important role in liver regeneration, and DNA synthesis occurs as early as 12 to 20 hours after partial hepatectomy. With the advent of cDNA microarray technology, which enables the screening of the genome-wide expression of thousands of genes, we were able to identify increased expression of angiotensinogen immediately after partial hepatectomy, with peaks at 6 hours and silencing after 24 hours. A recent study of liver regeneration using cDNA microarrays also demonstrated a 5.0-fold increment in angiotensinogen gene expression [28]. IL-6 is critically involved in liver regeneration after partial hepatectomy; however, the role of IL-6 during liver regeneration is not well defined. Aldeguer et al. characterized a paracrine mechanism by which cells of bone marrow origin, most likely Kupffer cells, regulated the regenerative capacity of hepatocytes through IL-6 expression [29]. Gewiese-Rabsch et al. demonstrated that IL-6 trans-signaling via soluble IL-6R was important for the physiological response of the liver to CCl4-induced chemical damage [26]. Kawasaki et al. reported that platelets promoted liver-specific endothelium cell proliferation and induced IL-6 and vascular endothelial growth factor production. IL-6 from liver-specific endothelium cells induced the proliferation of parenchymal hepatocytes [30]. We found that recombinant mouse IL-6 directly interacted with hepatocytes. The signaling pathways involved in angiotensinogen regulation were JAK/STAT3 and JAK/p38/NF-kB. Ray et al. demonstrated that STAT3 NH2-terminal acetylation was activated by the hepatic acute-phase response, and the acetylation of STAT3 was required for angiotensinogen induction by IL-6 [31].

The role of angiotensinogen in the regulation of blood pressure and water homeostasis is well recognized. However, the role of angiotensinogen in response to partial hepatectomy is not well studied. The major source of circulating angiotensinogen is the liver, and the plasma concentration of angiotensinogen is the rate-limiting step in the rennin-angiotensin system. Angiotensinogen serves as a reservoir for angiotensin I cleaved from the N-terminal by renin; angiotensin I is then converted into angiotensin II. The multifunctional effects of angiotensin II are mediated by activation of At1 or G protein-coupled receptors [32]. In a kidney-specific angiotensinogen knockout mouse model, the presence of liver-derived angiotensinogen suggested that the mechanism of kidney disease was due to the primary source of renal angiotensinogen protein and angiotensin II [33]. The upstream regions for the regulatory motifs of angiotensinogen include acute-phase responsive elements and glucocorticoid, estrogen, cAMP, and heat shock responsive elements [34]. NF-kB expression, known to increase during liver regeneration, is also a potent inducer of angiotensinogen by binding to the acute-phase responsive elements of the angiotensinogen promoter [35]. Within 1 hour of partial hepatectomy, there is evidence of NF-kB activation, which regulates signaling in cell cycle events, inflammation, and apoptosis [36]. Our results indicate that the activation of angiotensinogen occurs in the phase of early-delayed gene activation. It is plausible that angiotensinogen expression increases after NF-kB activation. In addition, the activation of NF-kB depends on tumor necrosis factor-α (TNF-α) during liver regeneration [1], [2]. TNF-α, a key regulator of liver regeneration, plays a dual role by inducing either hepatocyte proliferation or apoptosis during regeneration. TNF-α induction of cell apoptosis requires *de novo* angiotensin II generation [20]. Additionally, TNF-α may crosstalk with the rennin-angiotensin system in hepatocytes by enhancing the expression of key genes in the renin-angiotensin system, including angiotensinogen [33]. These lines of evidence support the notion that the angiotensinogen signaling pathway plays an important role in TNF-α/NF-kB-mediated liver regeneration.

In conclusion, we found that during liver regeneration, IL-6-enhanced angiotensinogen expression was dependent on the JAK/STAT3 and JAK/p38/NF-kB signaling pathways. The molecular mechanisms of angiotensinogen regulation may be targeted for therapeutic strategies preventing angiotensin II-related, ischemia-reperfusion injury of liver regeneration. Nevertheless, the exact role of angiotensinogen in liver regeneration requires further study.
